# The influence of olfactory stimuli on social cognition, prejudice and emotions: a systematic review of the literature

**DOI:** 10.3389/fpsyg.2026.1883832

**Published:** 2026-07-22

**Authors:** Michela Alemanno, Simone Migliore, Simona Massimino, Francesca Ferraioli, Andrea Schito, Carmelo M. Vicario, Sara Invitto, Giuseppe Curcio

**Affiliations:** 1 Department of Biotechnological and Applied Clinical Sciences, University of L'Aquila, L'Aquila, Italy; 2 Department of Cognitive Psychological Pedagogical and Cultural Studies Sciences, University of Messina, Messina, Italy; 3 Department of Biological and Environmental Sciences and Technologies, University of Salento, Lecce, Italy

**Keywords:** chemosignals, disgust, emotions, implicit bias, olfactory perception, prejudice, social cognition

## Abstract

This review synthesizes research on how olfactory stimuli influence social cognition, emotions, and prejudice, an area that remains understudied despite the olfactory system's direct neural connections to limbic regions like the amygdala and hippocampus. Following PRISMA 2020 guidelines, the authors systematically searched PubMed and Web of Science (2000–2025), identifying 21 eligible studies from 569 records. The literature reveals three core mechanisms through which odors exert their effects: (1) early perceptual modulation of social stimuli, (2) affective priming via disgust and valence systems, and (3) higher-order modulation of social judgments and moral evaluation. Unpleasant odors (e.g., sweat, rotten smells) increase vigilance, bias emotion perception, and amplify both implicit and explicit prejudices against marginalized groups such as gay men or immigrants. Putative human chemosignals like androstadienone and hedione subtly modulate mood, attention, and social judgments in context-dependent ways. Individual differences—including disgust sensitivity, neuroticism, and religiosity—systematically moderate these effects. Neuroimaging (EEG/fMRI) shows that odors engage distributed networks involving the amygdala, insula, and frontal regions, affecting early and late-stage emotional processing. The authors conclude that olfactory stimuli act as powerful, often subconscious modulators of social cognition. They propose that odors function as contextual priors that bias social inference, especially under conditions of ambiguity or uncertainty. These findings highlight the need to integrate olfactory dimensions into models of social behavior and bias formation. Future research should employ ecologically valid paradigms such as virtual reality to better understand how smells shape real-world interpersonal and intergroup dynamics.

## 1 Introduction

Olfaction, often considered the most ancient and visceral of the senses, plays a profound role in human social life. The olfactory system has direct neural pathways to brain regions central to emotion and memory, such as the amygdala and the hippocampus (Herz, 2009; Zald and Pardo, 1997). This unique neuroanatomy positions smell as a potent channel for

chemosignals, potentially influencing our judgments, emotions and social behaviors often outside of conscious awareness (Lundström and Olsson, 2005). Importantly, this privileged access to limbic circuitry suggests that olfactory inputs may shape social evaluation at both automatic and inferential levels.

In recent decades, a growing body of research has begun to systematically investigate how odors serve as invisible social stimuli. The conceptual framework often draws from the concept of the “Behavioral Immune System” (BIS) (Schaller, 2011), which proposes that humans have evolved psychological mechanisms to detect and avoid pathogens. As body odors can be cues to disease, this system may manifest in social contexts as prejudice or avoidance of out-groups perceived to have different hygiene practices (Zakrzewska et al., 2019; Stevenson et al., 2010). Within this framework, disgust emerges as a central affective mechanism linking sensory input to social evaluation. Notably, converging evidence suggests that disgust operates as a transdiagnostic construct across clinical conditions (Culicetto et al., 2023) and plays a broader role in shaping both affective and social processing (Vicario et al., 2018, 2022).

Concurrently, research into human chemosignals has explored whether substances like androstadienone or hedione can act as modulators of mood, attention and social perception, although the evidence remains contentious (Hummer and McClintock, 2009; Oleszkiewicz et al., 2021).

The influence of odor extends beyond disease avoidance and potential pheromones. Ambient smells can create an emotional context that biases how we perceive facial expressions and bodily emotions (Seubert et al., 2010; Li and Wang, 2021). Furthermore, experimentally induced disgust, often through foul odors, has been shown to heighten moral condemnation and amplify social prejudices, particularly against marginalized groups (Inbar et al., 2012; Cunningham et al., 2013). This suggests that emotions evoked by smell can readily “spill over” into social and moral judgments. Consistent with this view, neurophysiological evidence indicates that moral emotions such as indignation can directly modulate motor system excitability, including suppression of the tongue motor cortex, highlighting a tight coupling between affective evaluation and embodied responses (Vicario et al., 2022). Moreover, internal bodily states such as hunger and satiety have been shown to systematically influence moral judgment, further supporting the idea that visceral signals—including those conveyed by olfaction—interact with higher-order social cognition (Vicario et al., 2018).

### 1.1 Defining Olfactory influences on social cognition

Before proceeding, it is essential to clarify what we mean by “olfactory influences on social cognition.” In this review, we define this construct as the range of processes through which olfactory stimuli—whether consciously perceived or not—modulate the perception, interpretation, evaluation, and behavioral response to social agents and social situations. This encompasses several interrelated but conceptually distinct phenomena: (a) the impact of ambient odors on emotional perception and recognition (e.g., how a pleasant or unpleasant smell alters the interpretation of facial or bodily expressions); (b) the role of body odors and chemosignals in shaping social evaluations and interpersonal attitudes (e.g., how the smell of sweat influences prejudice toward outgroups or judgments of attractiveness); (c) the influence of olfactory cues on moral and attributional judgments (e.g., how an aversive smell associated with a person biases subsequent evaluations of their behavior); and (d) the modulation of social behavior by putative pheromones (e.g., how androstadienone or hedione affect attention, mood, or social perception). While these phenomena have often been studied in relative isolation, we argue that they share common underlying mechanisms, including direct limbic activation, affective priming, and crossmodal integration, and that integrating them within a unified theoretical framework can elucidate the full scope and complexity of olfactory influences on social life.

### 1.2 Integrated theoretical framework

The diverse phenomena reviewed here have typically been investigated through distinct theoretical lenses. Research on disgust and prejudice has drawn heavily on the Behavioral Immune System framework (Schaller, 2011), while studies on chemosignals have been informed by endocrinological and neurobiological perspectives (Hummer and McClintock, 2009; Oleszkiewicz et al., 2021), and work on odor-emotion interactions has been grounded in affective priming and crossmodal integration models (Seubert et al., 2010; Li and Wang, 2021). These areas are closely interrelated. At a neuroanatomical level, all olfactory influences on social cognition are mediated by the unique connectivity of the olfactory system with limbic and prefrontal regions. At a psychological level, they converge on common processes of affective evaluation, attentional prioritization, and associative learning. At a functional level, they serve adaptive roles in navigating the social environment—detecting threats, facilitating bonding, and guiding approach-avoidance behavior.

By integrating these areas within a single framework, this review aims to achieve three objectives. First, to identify shared mechanisms that cut across distinct domains, such as the role of the amygdala in emotional appraisal or the influence of disgust on evaluative judgment. Second, to highlight how these mechanisms interact—for instance, how individual differences in disgust sensitivity (a BIS-related trait) moderate responses to both ambient odors and putative pheromones. Third, to reveal gaps and inconsistencies in the literature that become apparent only when these areas are considered together, such as the contrasting patterns of replicability observed for pheromone effects vs. odor-induced prejudice effects. This integrative approach, we argue, offers a more complete and nuanced understanding of how the olfactory environment shapes our social world than any single perspective could provide.

Despite these advances, critical questions remain about the neural mechanisms and the interplay between olfactory cues, proxemic behavior (interpersonal distance) and implicit biases, especially in ecologically valid settings. In particular, current accounts remain fragmented across perceptual, affective, and social levels of analysis, lacking an integrative framework. A pioneering study protocol by Invitto et al. (2025) aims to address this gap by investigating the interaction between olfactory perception, propensity, and implicit or explicit prejudices through three experimental protocols conducted within a metaverse environment. This research employs virtual reality (VR) to examine how different odor (including putative pheromones and sweat) influence proxemic behavior when interacting with avatars from stigmatized social groups (e.g., based on race, sexual orientation, or body shape). Furthermore, the protocol integrates electroencephalography (EEG) to uncover the neural correlates of these prejudice-related responses and uses the Implicit Association Test (IAT) to measure implicit biases modulated by olfactory cues outside the VR environment (Invitto et al., 2025). This comprehensive approach highlights the potential of VR and olfactory stimuli as innovative tools for studying the unconscious mechanisms of bias formation in controlled, yet ecologically rich, scenarios.

The present systematic review aims to synthesize the existing empirical literature on how olfactory stimuli influence social cognition, prejudice, and emotions. By analyzing and integrating findings across experimental paradigms and levels of analysis, this review aims to identify core mechanisms through which the olfactory inputs shape our social perception, affective processing, and intergroup evaluation, ultimately providing a unified framework for understanding how the olfactory environments structure our social world.

## 2 Methods

The current systematic review was carried out in accordance with the guidelines and principles outlined by the Preferred Reporting Items for Systematic Reviews and Meta-Analyses (PRISMA) statement 2020 and checklist [12] (Page et al., 2021).

### 2.1 Search strategy and study selection

We searched on PubMed and Web of Science databases for studies published between January 2000 and December 2025. This time window was selected to capture the emergence of systematic research on olfaction and social cognition. The search was based on the following search string: (odor^*^ OR smell OR olfactory) AND (prejudic^*^ OR bias OR stereotyp^*^ OR “social perception”) AND (emotion^*^ OR affect^*^ OR disgust).

### 2.2 Inclusion and exclusion criteria

We included empirical studies investigating the effects of olfactory stimuli on social cognition, prejudice, or emotional processing.

In the first step (identification phase), we excluded duplicates, articles not written in English, reviews, systematic reviews, meta-analysis, and animal studies. In the second step (screening phase), we excluded studies that did not specifically investigate changes in social cognition or emotions. In the final inclusion phase, we retained studies reporting measurable psychological or behavioral outcomes associated with olfactory stimuli.

### 2.3 Data extraction and analysis

PRISMA recommendations for systematic literature analysis have been strictly followed. Given the methodological diversity of the included studies, no single standardized risk-of-bias instrument was considered appropriate across all included studies. Instead, study quality was assessed through a structured and reproducible qualitative framework: each study was independently evaluated by two reviewers according to predefined domains (sample characterization and selection bias in recruitment, assessment validity, statistical control, incomplete outcome data, and reporting transparency). Discrepancies were resolved through discussion and consensus; studies rated as methodologically weak were retained for completeness but interpreted with caution in the qualitative synthesis. Greater interpretative emphasis was placed on studies with stronger methodological characteristics.

## 3 Results

Of the 569 studies identified through database searching, 21 articles met the inclusion criteria and were included in the final analysis.

**Figure 1:**
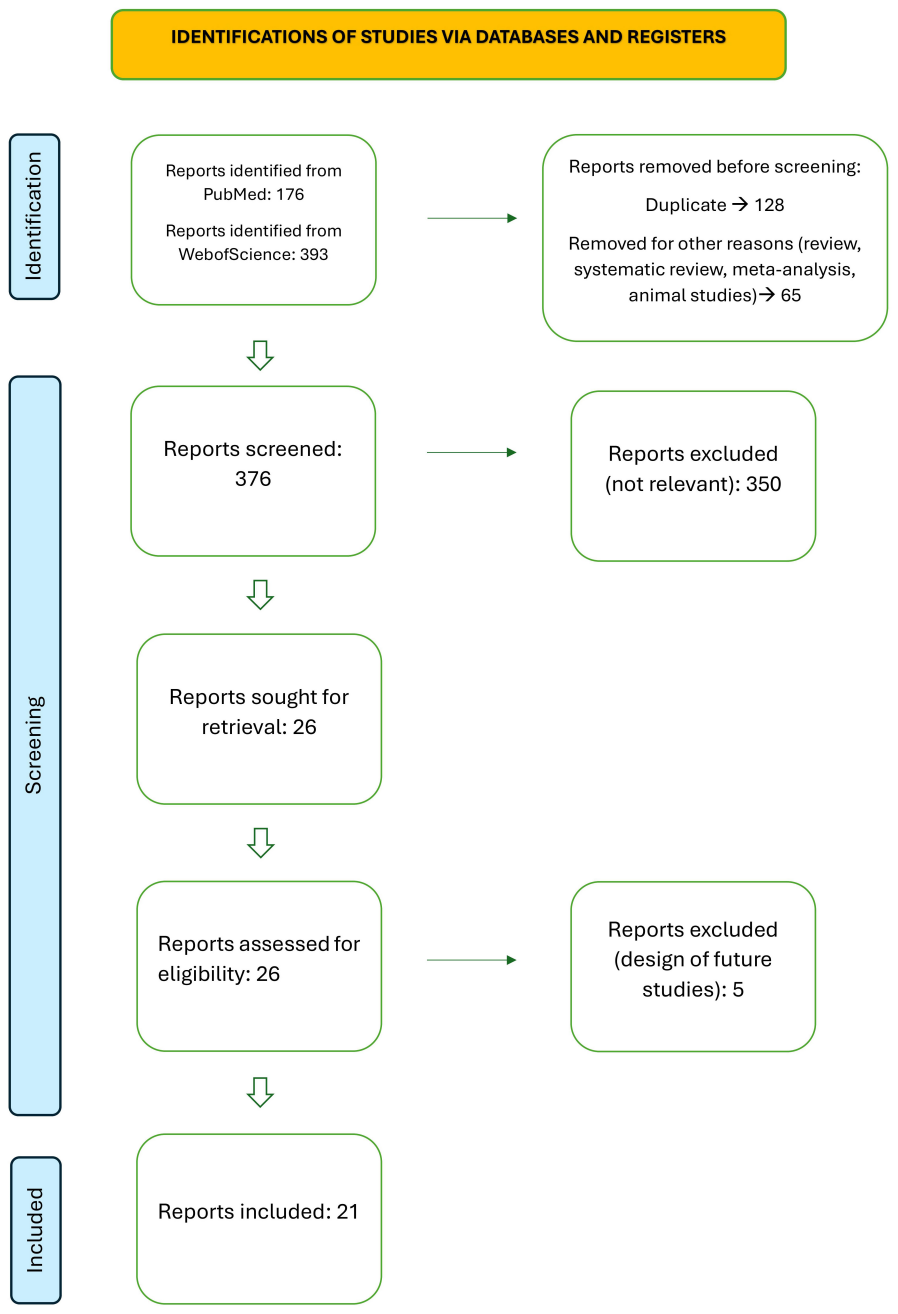
PRISMA 2020 flow diagram illustrating the study selection process. The diagram shows the identification, screening, and inclusion phases. A total of 569 records were identified through database searching (PubMed and Web of Science). After removing duplicates and excluding articles not written in English, reviews, systematic reviews, meta-analyses, and animal studies, 376 articles remained for screening. Following screening, 26 studies were assessed for eligibility. After excluding study protocols and pre-registrations without empirical data, 21 studies were included in the final qualitative synthesis.

Literature analysis and selection are summarized in a PRISMA-like diagram (Figure 1). Each study was analyzed, and a summary is reported in Table 1. Summary tables were structured to capture key study characteristics, including participant features, cognitive domains assessed, experimental paradigms, measurement techniques, and principal findings.

**Table 1:** Key characteristics of the 21 articles selected from the databases.

References	Participants	Cognitive domains assessed	Stimuli	Type of measure	Methodological comments	Results
Oleszkiewicz et al. (2021)	17 women	Social perception	Olfactory stimuli (odors) Visual stimuli (images)	Behavioral	Comparisons within group	The pheromone (Hedione) did not produce behavioral effects consistent in this context. The study suggests Hedione is not a potent human pheromone, at least in terms of influencing social perception of male faces.
Cecchetto et al. (2019)	28 women M age = 23.7 ± 4.2 years	Social cognition	Olfactory stimuli	Behavioral and functional (fMRI)	Comparisions within group	No difference was found in the moral choices themselves (deontological vs. utilitarian) based on the odor. The masked body odor changed brain activity. It specifically increased activity in areas for social cognition (supramarginal gyrus, angular gyrus) when people were processing complex, real moral dilemmas. Body odors make the brain process moral situations as more socially and emotionally salient, even if the final decision doesn't change and the smell is not consciously noticed.
Li and Wang (2021)	69 healthy adults 33 females; mean (*M*) age ± standard deviation (*SD*), 22.29 ± 2.56; range, 18–30 years	Cognitive processing of emotional visual stimuli, attention, perception	Visual Stimuli: emotional Body Language (EBL) Images Olfactory Stimuli: odor Contexts	Behavioral and electrophysiological (EEG)	Comparisons within group	Unpleasant odor context **enhanced recognition** of bodily expressions compared to pleasant or neutral odor contexts. The effects of odor on bodily expression processing occurred in two distinct phases: 150–200 ms post-stimulus VPPamplitudes were **greater** in the **unpleasant odor context** than in the pleasant one. After 200 ms there was an interaction between odor type and bodily expression. This reflects a **mood coherence effect**, where congruent emotional cues modulate neural responses.
Chen and Dalton (2005)	75 young adults 37 women *M* age (*SE*) = 23 (0.77) 38 men *M* age (*SE*) = 24 (0.79)	Attention and Perceptual Processing	Emotion induction stimuli Olfactory stimuli	Behavioral	Comparisions between and within group	Women detected pleasant smells faster than neutral smells. Men perceived all smells as more intense when they were in an emotional state (happy, sad, or hostile) compared to a neutral state. Anxious women rated emotional smells as stronger than the neutral smell. Anxious men detected emotional smells faster than the neutral smell, while calm men did not. Personality did not have a direct overall effect, but it modulated how people responded to emotional vs. neutral smells. Everyone, regardless of group, rated the unpleasant odor as more intense than the pleasant or neutral ones.
(Syrjänen et al. 2018a,b)	40 partecipants 22 females (*M* age = 26.2; *SD* = 6.82)	Visual attention	Visual stimuli (facial expression) Olfacotry stimuli	Behavioral	Comparisions within group	While odors clearly affect emotional evaluations of faces (valence and arousal), they do not modulate visual attention to those faces in the dot-probe task. The unpleasant odor may activate the behavioral immune system, leading to increased vigilance over time.
(Syrjänen et al. 2018a,b)	58 participants	Social perception	Visual and olfactory stimuli	Behavioral and electrophysiological (EEG)	Comparisions within group	The study found that background smells create a general “mood” that biases how we judge faces (valence). Furthermore, smells specifically interact with certain emotions, making happy faces seem less exciting in a bad smell context and changing the very early brain activity involved in processing disgusted faces.
(Cunningham et al. 2013)	146 partecipants (51 male) *M* age = 18.8; *SD* = 1.4	Social cognition	Visual stimuli Olfactory stimuli	Behavioral	Comparisons between groups	Participants in the “body odor” condition looked at images of gay couples for a shorter time compared to straight couples than participants in the other conditions. This indicates implicit avoidance or discomfort specific to gay men. On the “feelings thermometer,” participants in the “body odor” condition reported colder feelings toward gay men relative to straight men, compared to the control group. For lesbian couples, the odor itself didn't cause a general bias. However, in the “body odor” condition, individuals with a higher sensitivity to sexual disgust rated lesbian couples as less pleasant compared to straight couples.
(Kovács et al. 2004)	62 male (*M* age = 29.2 years)	Visual perception	Visual stimuli Odorants	Behavioral and functional (fMRI)	Comparisions between and within group	Passive inhalation of sex hormone-like compounds (androgen and estrogen) can bias men's visual gender judgments. The effect is direction-specific, occurring only when female faces are masculinised. This suggests a cross-sensory modulation at the level of gender-specific facial attribute processing, possibly involving the fusiform gyrus.
(Seubert et al. 2010)	21 males mean age 23.95 (*SD* = 2.25) 23 females mean age 24.83 (*SD* = 5.27)	Emotion recognition	Visual stimuli Olfactory stimuli	Behavioral	Comparisions within group	This study found that presenting an odor just before a picture of a face **speeded up people's recognition of disgusted facial expressions**. Reaction times for identifying disgusted faces were significantly faster when preceded by any odor compared to the no-odor condition. This facilitation effect was **specific to disgust**; it did not occur for happy or neutral faces.
(de Groot et al. 2021)	31 female (Mage = 23.23 years)	Social Perception and Emotional Cognition	Visual stimuli Olfactory stimuli	Behavioral	Comparisions within group	All three doses of fear sweat (Low, Medium, and High) were equally effective at biasing participants to perceive ambiguous faces as more fearful compared to neutral sweat. Sniffing any dose of fear sweat led to similar, fear-specific increases in sniff duration (a sign of sensory vigilance) compared to neutral sweat.
(Gaby and Dalton 2019)	48 females (mean age 27.5 5.68)	Learning and memory	Olfactory stimuli Visual stimuli Somatosensory stimuli	Behavioral	Comparisions between and within group	Participants successfully learned to associate a specific individual's body odor with a negative event (electric shock). This was shown by a significantly increased galvanic skin response (GSR) when they smelled the shock-paired odor, even when no shock was administered. The learning effect was equally strong for both natural body odors and perfumed body odors. This indicates that perfume does not mask the underlying individual signature of a person's body odor; participants could still tell two people apart even when they wore the same fragrance. The conditioned body odor influenced social judgment. When smelling the shock-paired odor, participants rated neutral faces as significantly more surprised than when smelling the control odor. This suggests a learned olfactory cue can bias how we interpret visual social information.
Lundström and Olsson (2005)	37 women (*M* age = 25.35 years *SD* = 4.63)	Sustained attention	Olfactory stimuli Visual stimuli	Behavioral	Comparisons between groups	Androstadienone can modulate mood and arousal, but not behavior or perception of attractiveness. Social context, specifically the sex of the experimenter, plays a crucial role in whether these effects manifest.
(Hornung et al. 2018)	73 participants (46 women) mean age = 24.33 years (*SD* = 3.35)	Cognitive Control and Interference Processing	Olfactory stimuli Visual and cognitive stimuli	Behavioral	Comparisions between and within group	The study found **no evidence** that a person's genetic profile or their sex/hormonal group influenced how they responded to AND. The researchers provided **strong quantitative evidence** in favor of the null hypothesis. There is no modulating effect of genotype or sex on AND's action. A single, small effect of AND was found: participants made **fewer errors** in the Stroop task during AND exposure compared to placebo, regardless of their group or genotype. There was no effect on reaction times. Some correlations were found between hormone levels and task performance, but these were inconsistent and differed between groups and odor conditions. The authors concluded these were not reliable evidence that AND modulates the effects of sex hormones.
(Lu et al. 2024)	163 participants (92 females; mean age = 22.60, s.d. = 2.92)	Decision making	Decision making stimuli Olfactory stimuli	Behavioral	Comparisions between groups	Exposure to androstadienone did **not** make males more generous, nor did it make females more selfish, compared to the placebo group. The chemosignal did **not** alter how participants perceived their social closeness to others (e.g., family, friends, strangers). Androstadienone did **not** affect the underlying cognitive decision process, such as initial bias toward generosity or the speed of evidence accumulation.
(Zakrzewska et al. 2020)	35 participants (*M* age = 28.5 years; *SD* = 6.29) 25 females	Social cognition	Visual stimuli Olfactory stimuli	Behavioral	Comparisions within group	This study found a clear link between sensitivity to disgusting body odors and unconscious prejudice. Higher **Body Odor Disgust Sensitivity (BODS)** was significantly associated with stronger **implicit bias** against the Romani outgroup. People more disgusted by body odors showed a greater automatic preference for their own group. The presence of a background **unpleasant, sweat-like odor** did **not** increase implicit bias, nor did it interact with participants' BODS levels.
(Homan et al. 2017)	17 participants (9 female) mean age of 27.7 (3.9)	Social cognition	Olfactory and visual stimuli	Behavioral and functional (fMRI)	Comparisions within group	Masked body odors increase brain activity in social/emotional regions during moral dilemmas, but do not change final decisions. Aversive smell associations cause a shift in social reasoning style, making people more likely to blame a person's character (not their situation).
(Vieillard et al. 2021)	40 older adults aged 65–80 (*M* = 70.23, *SD* = 4.14; 52.5% females) 38 younger adults aged 20–35 (*M* = 24.8, *SD* = 3.78, 57.8% females)	Emotional processing	Olfactory stimuli	Behavioral	Comparisions between group	This study found that compared to younger adults, older adults rated unpleasant odors as significantly less unpleasant. There was no significant age difference in the ratings for pleasant odors. This shows that older adults have a “positivity effect,” perceiving negative smells in a less negative light. In younger adults, the ratings for hedonic valence (pleasantness) and emotional intensity were only weakly related. In older adults, these two dimensions were strongly correlated, meaning they did not distinguish as clearly between the pleasantness and the intensity of an odor. This “blending” of emotional dimensions is known as emotional dedifferentiation. In short, as people age, their emotional response to smells becomes less negative and more simplified, with pleasantness and intensity becoming more intertwined.
(Zakrzewska et al. 2019)	805 participants 444 females (mean age=37.81, *SD* = 11.62)	Social cognition	Olfactory stimuli	Behavioral	Comparisions between groups	This study found that an individual's sensitivity to disgusting body odors is linked to prejudice against immigrants. Higher **Body Odor Disgust Sensitivity (BODS)** was significantly associated with more negative attitudes (xenophobia) toward a fictional group of African refugees. People with higher BODS were more likely to perceive the refugee group as having different hygiene and food preparation practices, which in turn predicted more negative attitudes toward them. The link between BODS and prejudice was specific to the fictional refugee group. The connection between BODS and general opposition to immigration was much weaker and inconclusive.
(Mutic et al. 2016)	31 participants 15 males (*M* = 27.80 years, *SD* = 8.83 years) 16 females (*M* = 29.56 years, *SD* = 9.33 years)	Social perception	Olfactory stimuli	Behavioral	Comparisions between and within group	Both male and female body odors were rated as more masculine than the neutral no-odor sample. Participants could distinguish between odors, best at telling female odors from the no-odor sample. Exposure to any body odor (male or female) made participants rate social stimuli as more feminine. Both male and female chemosignals caused attributes to be rated as more feminine compared to the no-odor condition.
Hummer and McClintock (2009)	50 subjects 20 men 30 women (mean±SEM: 20.9 ± 0.6)	Attention	Olfactory stimuli Visual stimuli	Behavioral	Comparisions within group	Androstadienone (AND) specifically enhanced attention to emotional information, but had no effect on general attention, neutral attention or attention to social information. AND helped sustain self-reported feelings of being Attentive and Sharp over the course of the experiment, compared to the control. However, it did not influence participants' positive or negative mood. The effects of AND on emotional attention were the same for both men and women.
(Inbar et al. 2012)	59 partecipants (50 women)	Social cognition	Olfactory stimuli	Behavioral	Comparisions between group	This study found that inducing a state of disgust in participants by exposing them to a noxious ambient odor caused them to report less warmth and liking specifically toward gay men compared to heterosexual men. This effect was specific to gay men. Furthermore, the effect was equally strong for both political liberals and conservatives, suggesting that even individuals who consciously reject disgust as a basis for moral judgment are susceptible to its influence.

### 3.1 Literature search

The initial search yielded 569 records. We had at that point 376 articles. Then (screening phase) we excluded studies that did not measure outcomes related to social cognition or emotions and/or investigations without olfactory stimuli and thus remained 26 studies. In the end, after excluding papers with only study protocols or pre-registration, a total of 21 articles were analyzed. After excluding study protocols and pre-registrations without empirical data, 21 studies were retained for qualitative synthesis.

### 3.2 Participant's characteristics

The included studies aimed to investigate the influence of social cognition, prejudice, and emotions. The 21 studies included in this review encompassed a total of approximately 1905 participants with sample sizes ranging from 17 (Oleszkiewicz et al., 2021) to 800 (Zakrzewska et al., 2019). The participants' samples were heterogeneous and primarily consisted of healthy adults from the general population and university students, covering a wide age range from late adolescence to older adulthood (e.g., 18–30 in Li and Wang, 2021; 65–80 in Vieillard et al., 2021).

In terms of demographic composition, the studies showed considerable variation based on their specific research questions. Some investigations focused on specific genders and sexual orientations; for instance, several studies recruited exclusively heterosexual women to investigate responses to putative male pheromones (Lundström and Olsson, 2005; Oleszkiewicz et al., 2021), while others involved only male participants for experiments on gender discrimination (Kovács et al., 2004) or as donors of chemosignals (de Groot et al., 2021). However, many other studies employed mixed-gender samples to ensure broader generalizability.

A rigorous screening process was commonly implemented to ensure data quality. Participants were typically required to have normal olfactory function, often verified using standardized tests such as the Sniffin' Sticks. Furthermore, individuals were excluded if they had neurological, psychiatric, or respiratory conditions that could potentially affect their sense of smell or interfere with task performance. In studies involving putative pheromones, female participants were sometimes tested during specific phases of their menstrual cycle (e.g., the periovulatory phase) to control for hormonal fluctuations (Oleszkiewicz et al., 2021; Lu et al., 2024).

Finally, some studies intentionally selected participants based on specific psychological traits to examine moderating factors. This included individuals with high or low scores on neuroticism and anxiety (Chen and Dalton, 2005), or those with high body odor disgust sensitivity (M. Z. Zakrzewska et al., 2020). This heterogeneity enables the examination of individual differences as moderators of olfactory effects on social cognition, and prejudice, although it may also contribute to variability across findings.

## 4 Neural bases of olfactory-social integration

A growing body of evidence uses neuroimaging (fMRI, EEG) to uncover when and where in the brain olfactory information interacts with the processing of social stimuli, consistently showing that this integration operates across multiple processing stages, from early perceptual encoding to higher-order evaluative processes. Importantly, these findings suggest that olfactory inputs act as contextual signals that bias the interpretation of social information rather than merely adding parallel sensory input.

### 4.1 Perceptual modulation by odors

Odors can prime the visual system, influencing the initial encoding of social signals like faces. Using EEG (Syrjänen et al. 2018b) demonstrated that background odors modulate early stage of face processing. In this experiment, the stimuli were: pictures of faces showing happy, disgusted, and neutral expressions; a pleasant odor (lilac) and an unpleasant odor (valeric acid, smells like sweat). A no-odor condition served as the control. They found that while odors globally biased the perceived pleasantness of all faces, a specific interaction emerged at the neural level: the N170, an early ERP component sensitive to faces, showed a larger amplitude for disgusted faces specifically in a pleasant odor context. This suggests that emotionally incongruent multisensory information (e.g., a pleasant odor paired with a disgusted face) increases early perceptual processing demand, likely reflecting prediction error or conflict at the perceptual level.

Complementing this, (Seubert et al. 2010) used fMRI to show that both pleasant and unpleasant odors facilitate the recognition of disgusted facial expressions. They used three olfactory conditions: vanillin (pleasant), Hydrogen Sulfide (H_2_S, rotten egg smell; unpleasant), and ambient air (neutral baseline). Odors were briefly released into the room prior to the appearance of visual stimuli, during which participants were required to identify facial expressions as quickly as possible. Concurrent fMRI acquisition measured the Blood-Oxygen-Level-Dependent (BOLD) signal as an index of neural activity. Results indicated that odor-facilitated recognition of disgusted faces was associated with a neural network encompassing both modality-specific regions (anterior insula) and modality-unspecific regions (fusiform gyrus), suggesting the existence of a dedicated system for cross-modal emotional integration.

Together, these findings suggest that olfactory inputs can bias early visual processing both through congruency effects (facilitating matching emotional signals) and incongruency effects (increasing processing demands when signals conflict).

Li and Wang (2021) extended these findings beyond facial expressions to emotional body language (EBL), using ERPs to investigate how odors influence the recognition of happy, fearful, and neutral bodily expressions. As stimuli, they used images of bodily expressions (from the BEAST database) showing happy, fearful, or neutral postures and odor stimuli: pleasant (lemon scent), unpleasant (rotten fish scent), neutral air (no odor). Odors were diffused naturally in the room using a diffuser to simulate real-life conditions. Participants sat in a room with one of the odor contexts, and they viewed images of bodily expressions on a screen; for each image, they had to quickly identify the emotion (happy, fearful, or neutral) by pressing a key. EEG (electroencephalography) was recorded to measure brain activity during the task. They identified a two-phase process: an early enhancement of emotional processing for all bodies in unpleasant contexts (150–200 ms, reflected in VPP amplitudes), followed by a later mood-congruency effect (>200 ms) where fearful bodies were processed with less neural effort (smaller N2 and LPP amplitudes), specifically in an unpleasant context. This suggests that olfactory inputs first act as a general amplifier of emotional salience and subsequently as a selective facilitator of congruent emotional information.

### 4.2 Modulation of emotional, cognitive, and moral processing

Odors also modulate more elaborate stages of social and emotional processing. At higher levels of processing, olfactory inputs appear to influence moral evaluation and social inference. Cecchetto et al. (2019) investigated whether subliminal social chemosignals modulate moral decision-making. Using fMRI, the authors exposed female participants to moral dilemmas paired with either a neutral masker odor or a masker containing non-consciously perceived male body odor. While the olfactory manipulation did not significantly alter behavioral choices (e.g., utilitarian vs. deontological responses), it significantly modulated neural activity. The presence of masked body odor selectively enhanced activation in brain regions associated with social cognition and empathy—specifically the left supramarginal gyrus during high-conflict (incongruent) dilemmas and the right angular gyrus during accidental harm scenarios. These findings suggest that olfactory signals can bias the neural processing of moral dilemmas even in the absence of explicit awareness, selectively engaging socio-emotional networks without necessarily altering overt moral choices.

Similarly, (Homan et al. 2017) demonstrated that associative learning involving olfactory stimuli can exert lasting effects on social judgment. They used fMRI to examine how pairing a person's face with a bad smell affects later social judgments about that person. Participants first associated one face with a foul odor (CS+) and another with nothing (CS–). Later, inside the scanner and with no smell present, they read stories about both people's behaviors and judged whether those behaviors reflected personality (trait) or circumstances (situation). The CS+ person's behaviors were more often attributed to their personality, regardless of whether the behavior was positive or negative. fMRI revealed reduced activity in the vmPFC and right Angular Gyrus specifically during attribution judgments for the CS+ person—regions known to support flexible, context-sensitive social reasoning. Notably, this effect was not observed with aversive visual conditioning, suggesting the degree of modality specificity. fMRI data suggests that olfactory conditioning slightly disrupts the brain processes involved in making nuanced social judgments., suggesting a degree of modality specificity. Overall, these findings indicate that olfactory conditioning can bias attributional processes, potentially reducing the flexibility of social inference mechanisms.

Taken together, these studies suggest that olfactory inputs influence higher-order social cognition through at least two complementary mechanisms: (i) implicit modulation of socio-emotional neural systems, even in the absence of conscious awareness, and (ii) associative learning processes that shape subsequent social judgments. These effects appear to operate primarily at the level of neural representation and inference, rather than directly altering overt behavioral responses, highlighting a dissociation between neural modulation and explicit decision-making.

## 5 The impact of odors on social judgments and prejudice

Beyond basic perception, odors influence complex social evaluations, from first impressions to deep-seated prejudices against social groups. Importantly, these effects appear to operate by shaping evaluative biases and interpretative frameworks rather than directly altering perceptual sensitivity.

### 5.1 Olfactory biases in social perception

Several studies confirm that ambient odors create an affective lens through which we evaluate others. (Syrjänen et al. 2018a) examined whether background odors influence attentional and evaluative processing of emotional facial expressions using a dot-probe paradigm. The results showed that odor valence influenced explicit face ratings in a global manner: all faces were perceived as more negative in the unpleasant odor context and more positive in the pleasant odor context, compared to the no-odor condition. Additionally, both pleasant and unpleasant odors increased the perceived arousal of faces relative to the neutral condition. However, odor condition did not reliably modulate spatial attention to emotional faces, as indexed by standard reaction time measures in the dot-probe task. Instead, a time-course analysis revealed that unpleasant odor sustained vigilance rather than initial attentional orienting. Overall, the study demonstrates that background odors shape the subjective experience of social stimuli in a valence-congruent manner without automatically biasing spatial attention, highlighting a dissociation between explicit affective ratings and implicit attentional processes. These findings indicate that olfactory inputs primarily influence evaluative appraisal rather than early attentional selection, acting as contextual affective biases rather than bottom-up attentional drivers.

Complementing these findings research has also demonstrated that human chemosignals subtly shape basic social perception. (Mutic et al. 2016), investigated whether body odors convey gender-related information and influence the interpretation of ambiguous social stimuli. In a double-blind design, healthy participants were exposed to male chemosignals, female chemosignals, or a no-odor control while rating gender-neutral faces and personality adjectives.

Although participants were unable to explicitly identify the sex of the odor donors—displaying a general tendency to perceive any body odor as “masculine”—the presence of chemosignals nevertheless biased their social judgments. Specifically, exposure to any body odor (male or female) led participants to rate gender-neutral adjectives as more feminine, and, particularly among female raters, to perceive neutral faces as more feminine. Importantly, these effects occurred without any concurrent change in self-reported mood, suggesting a direct, implicit influence on social cognition. The pattern reveals a dissociation between explicit categorization of olfactory stimuli and their implicit influence on social interpretation, indicating that chemosignals can bias social inference independently of conscious identification.

Overall, these findings suggest that human chemosignals act as implicit cues that modulate the interpretation of socially ambiguous information.

Research on body odors confirms they carry socially relevant information that we can learn from. (Gaby and Dalton 2019) demonstrated that individual body odor can become conditioned threat signals through associative learning. Using a differential conditioning paradigm, participants were exposed to the body odors of two different donors, with one odor (CS+) repeatedly paired with a mild electric shock and the other (CS-) presented without reinforcement. Results showed that participants successfully acquired the aversion, exhibiting significantly higher galvanic skin responses (GSR) to the shock-associated odor during later unreinforced trials, demonstrating discriminative learning between individual chemosignals. Crucially, this learning persisted even odors were masked by perfume, suggesting that individual olfactory signature remain behaviorally relevant.

Furthermore, this learned aversion “spilled over” to influence social perception in a different modality: neutral faces were rated as significantly more surprised when participants were exposed to the shock-associated odor compared to the control odor, consistent with heightened vigilance being misattributed to the ambiguous social stimuli. These findings indicate that olfactory learning generalized across modalities, biasing the interpretation of social signals even in the absence of the original conditioning context.

Taken together, these studies suggest that olfactory influences on social perception operate through at least three complementary mechanisms: (i) valence-driven modulation of evaluative judgments, (ii) implicit biasing of social inference independent of conscious awareness, and (iii) associative learning processes that can generalize across sensory modalities. Critically, these effects converge in shaping the interpretation of socially relevant, often ambiguous information, rather than directly altering perceptual sensitivity.

### 5.2 Odors, emotions, and prejudice

Chen and Dalton (2005) investigated how emotional state and personality traits jointly modulate olfactory processing. They induced emotional states (happy, sad, hostile, neutral) via video clips and measured detection speed and intensity ratings for pleasant, unpleasant, and neutral odors. Results revealed sex-specific effects: women detected pleasant odors faster than neutral ones, while men perceived odors as more intense when in any emotional state (happy, sad, or hostile) compared to neutral, particularly for unpleasant odors during hostile states. Personality modulated these effects: anxious women perceived emotional odors (both pleasant and unpleasant) as more intense than neutral odors, while neurotic and anxious men detected emotional odors faster. These findings indicate that olfactory processing is dynamically shaped by internal affective states and stable personality traits, suggesting a bidirectional interaction between emotion and olfactory sensitivity.

A powerful line of research demonstrates that disgust—whether induced by an odor or a pre-existing trait—can increase prejudice, particularly toward sexual minorities. (Cunningham et al. 2013) showed that exposure to a disgust-inducing body odor reduced visual attention to images of gay male couples (implicit bias) and increased negative explicit evaluations. This effect was specific to gay men and did not extend to lesbian couples. However, individual differences in sexual disgust sensitivity moderated explicit evaluations: participants with higher sexual disgust rated images of lesbian couples as less pleasant. These findings suggest that disgust, particularly when socially contextually, can selectively bias intergroup attitudes, with effects modulated by individual sensitivity to disgust. distinct patterns for gay men and lesbian women.

In a powerful demonstration of this phenomenon, (Inbar et al. 2012) showed that simply spraying a “stink spray” in a room was enough to cause participants to rate gay men, but not other social groups. In a between-subjects design, participants completed questionnaires in a room that either contained a noxious ambient odor (commercial “stink spray”) or no odor. Using a “feeling thermometer,” participants rated their warmth toward 19 different social groups, including gay men, lesbians, and heterosexual individuals. Results showed that participants in the smell condition reported significantly less warmth toward gay men (relative to heterosexual men) compared to those in the control condition. This effect was specific to gay men—it did not generalize to lesbians or other outgroups—and was equally strong across the political spectrum. These results highlight the capacity of incidental disgust to directly bias explicit social attitudes in a target-specific manner.

Correlational research supports these experimental findings by linking a stable personality trait—body odor disgust sensitivity (BODS)—to prejudice. (Zakrzewska et al. 2019) found that higher BODS was significantly associated with more negative attitudes (xenophobia) toward a fictional group of refugees (“Drashneean refugees”). This relationship was partially explained by the perception that the outgroup's hygiene and food practices were dissimilar, suggesting the behavioral immune system uses olfactory cues to signal potential threat from “different” others. However, (Zakrzewska et al. 2020) found that while BODS correlated with implicit bias against an outgroup (the Romani) using an Implicit Association Test, this bias was not increased by acute exposure to a sweat-like odor (valeric acid) compared to pleasant or neutral odors. This suggests that disgust sensitivity functions as a stable dispositional predictor of prejudice, whereas transient olfactory cues may not be sufficient to amplify such biases in the absence of contextual relevance.

Taken together, these findings converge on the idea that disgust represents a central mechanism linking olfactory processing to social evaluation. Specifically, olfactory cues appear to influence prejudice through three interacting pathways: (i) state-dependent modulation of affective processing, (ii) trait-like individual differences in disgust sensitivity, and (iii) context-dependent activation of the behavioral immune system. Importantly, these mechanisms suggest that olfactory inputs bias social evaluation in ways that depend on both internal states and the perceived relevance of potential social threat.

## 6 Putative human pheromones

A distinct but related body of work investigates compounds like androstadienone (AND) and Hedione, often called putative human pheromones. Overall, evidence for their social effects is mixed, with most findings pointing to subtle and context-dependent modulations rather than robust or automatic behavioral responses.

### 6.1 Evidence for context-dependent effects

Some studies report positive behavioral findings. Oleszkiewicz et al. (2021) found that exposure to Hedione increased women's explicit ratings of male facial attractiveness and likeability, without affecting their mood or the speed of face recognition in an implicit task. This behavioral dissociation—an effect on conscious evaluation but not automatic processing—suggests that Hedione may preferentially influence conscious evaluative processes rather than automatic perceptual mechanisms.

(Hummer and McClintock 2009) provided a nuanced behavioral picture, showing that AND acts as a precise modulator of emotional attention. In a series of four studies, AND accelerated reactions to emotional faces in a dot-probe task and slowed down color-naming for emotional words in an emotional Stroop task, but had no effect on attention to non-emotional or non-social stimuli. This pattern of behavioral results indicates AND specifically tunes the brain to prioritize emotionally salient information, supporting the Emotional Attention Hypothesis. AND also attenuated the normal decline in self-reported focus over time, a subjective behavioral effect, without directly altering mood. (Lundström and Olsson 2005) demonstrated that the effects of AND on women are critically dependent on social context. In a double-blind study, women exposed to AND showed enhanced mood and physiological arousal (finger pulse, skin temperature), but only when the experimenter was male. No effects were observed on sustained attention or ratings of male facial attractiveness. These findings highlight that AND effects are contingent on social context rather than reflecting fixed stimulus-driven responses.

(Kovács et al. 2004) provided evidence for cross-modal sensory integration by showing that chemosensory cues can bias visual gender discrimination in faces. Compared to a neutral control (water), men exposed to androgen (a male-associated steroid) required less masculinization to judge a face as male, while those exposed to estrogen required more masculinization. These effects were specific to masculinization trials, indicating that chemosensory signals can influence categorical judgments when visual information is uncertain.

Taken together, these findings suggest that putative human chemosignals may influence social cognition through several limited and context-dependent mechanisms: (i) modulation of explicit evaluative judgments, (ii) selective tuning of attention toward emotionally salient stimuli, and (iii) context-sensitive effects that depend on social and environmental conditions.

### 6.2 Evidence for null or inconsistent effects

In contrast, several studies have failed to find significant effects. (Lu et al. 2024) conducted a rigorous, incentive-compatible study and found no effects of AND on prosocial behavior (generosity) in a social distance-dependent task, nor on perceived social closeness, even after controlling for conscious detection of the compound. Additionally, AND did not alter perceptions of social closeness to others (family, friends, strangers).

(Hornung et al. 2018) similarly found limited and inconsistent effects of AND on mood, perception, or cognition across 80 participants tested in a within-subjects design (AND vs. placebo). These effects were not modulated by genetic variations in the putative AND receptor (OR7D4), participant sex, or hormonal status (testosterone, progesterone, estradiol levels). This suggests that individual differences in response to AND are not easily explained by these biological factors and that AND does not reliably modulate behavior or perception based on them.

Studies on other chemosignals also show limitations. (de Groot et al. 2021) investigated whether humans communicate the intensity of fear through body odor. While they found that fear sweat biased women toward perceiving fear in ambiguous faces and induced adaptive sniffing changes (longer sniff durations) compared to neutral sweat, this effect was “dose-invariant”—low, medium, and high fear sweat produced similar effects, despite all samples being rated as equally intense and pleasant. This suggests an all-or-none alarm response rather than a graded signal. Interestingly, the left amygdala showed the highest responses within a specific bandwidth of fear intensity, and amygdala activity correlated with the percentage of faces perceived as fearful, demonstrating subconscious chemosignal processing.

Taken together, these findings highlight important boundary conditions for the effects of putative human chemosignals: (i) limited or absent behavioral effects in controlled settings, (ii) lack of consistent modulation by proposed biological factors, and (iii) non-linear or non-graded responses to socially relevant chemosignals.

## 7 Age-related changes in olfactory emotional processing

Beyond the immediate effects of odors on social cognition, emerging research highlights how individual factors such as age modulate the very experience of smells. (Vieillard et al. 2021) investigated age-related differences in emotional responses to odors, comparing younger (20–35 years) and older adults (65–80 years) on ratings of valence, intensity, and familiarity for 50 odorants.

Results revealed a reduced negativity bias with age: older adults rated unpleasant odors as significantly less unpleasant than younger adults, while pleasant odors were rated similarly across groups. Furthermore, older adults showed emotional “dedifferentiation,” with a stronger and simpler coupling between pleasantness and intensity ratings. Familiarity also played a greater role in shaping hedonic experience for older adults. These findings suggest that aging is associated with a shift toward reduced negative reactivity and simplified affective encoding of olfactory stimuli, alongside an increased reliance on familiarity. Importantly, such changes may have downstream consequences for social cognition, as reduced sensitivity to aversive olfactory cues could attenuate disgust-related responses and, consequently, modulate the expression of prejudice and avoidance behaviors across the lifespan.

Taken together, these findings suggest that age-related changes in olfactory processing may influence social cognition through: (i) reduced sensitivity to unpleasant stimuli, (ii) simplified affective encoding, and (iii) increased reliance on familiarity-based evaluations.

## 8 Discussion

This systematic review synthesized evidence from 21 studies to elucidate the complex role of olfactory stimuli in modulating social cognition, prejudice, and emotions. Overall, the findings indicate that olfactory cues can influence social cognition in subtle, often implicit, and context-dependent ways, operating through multiple psychological and neural pathways.

To interpret these findings, it is useful to situate them within complementary theoretical frameworks, beginning with the Behavioral Immune System theory (Schaller, 2011), which conceptualizes a set of psychological mechanisms evolved to detect and respond to potential pathogens. The present evidence is broadly consistent with this framework. Unpleasant odors, especially those associated with the human body (such as sweat or valeric acid) or decay, consistently activate disgust responses that translate into negative social evaluations. In line with BIS logic, this activation does not remain confined to sensory experience but extends to influence complex social judgments: we have seen how unpleasant odors amplify vigilance (Syrjänen et al., 2018b; Li and Wang, 2021), bias the perception of emotional expressions toward negativity (Syrjänen et al., 2018a), and, most critically, exacerbate both implicit and explicit prejudices against sexual minorities (Inbar et al., 2012; Cunningham et al., 2013) and immigrants (Zakrzewska et al., 2019). Importantly, these effects do not appear to be uniform but rather depend on contextual relevance and individual differences.

The fact that dispositional traits such as Body Odor Disgust Sensitivity (BODS) predict higher levels of xenophobia (Zakrzewska et al., 2019) further strengthens this interpretation, pointing to stable individual variability in the sensitivity of these mechanisms.

A second relevant theoretical framework is Embodied Cognition (Barsalou, 2008), which assumes that cognitive processes are grounded in the body's sensorimotor experiences. Within this perspective, emotional processing is closely linked to bodily and interoceptive states. Within this perspective, emotional processing is closely linked to bodily and interoceptive states, rather than reflecting purely abstract representations. In line with this perspective, recent evidence indicates that internal physiological states can directly modulate social evaluation. For instance, transient interoceptive conditions such as hunger have been shown to influence implicit sexual prejudice, suggesting that disgust-related avoidance mechanisms are dynamically shaped by the organism's physiological state rather than being solely driven by external sensory cues (Lucifora et al., 2025).

The neuroscientific evidence emerging from this review supports this view. Neuroimaging findings indicate that olfactory stimuli engage a distributed network-including the amygdala, insula, and frontal regions–implicated in affective and social processing (Seubert et al., 2010). The anterior insula, in particular, is a key region for the representation of visceral states and is activated both during the direct experience of disgust and during the observation of disgusted facial expressions—an emotional mirroring mechanism that supports embodied intersubjectivity. Complementing this, ERP studies reveal that this integration occurs across distinct temporal phases: an early modulation (150–200 ms, VPP) reflecting a general enhancement of emotional processing in unpleasant olfactory contexts, followed by later mood-congruency effects (>200 ms, N2, LPP) indicating facilitated processing when the odor and visual stimulus share the same emotional valence (Li and Wang, 2021). This two-phase pattern suggests that odor first acts as a non-specific “amplifier” of emotional attention, and subsequently as a specific “modulator” that facilitates the processing of congruent stimuli—a mechanism that optimizes social information processing based on emotional context.

**Figure 2:**
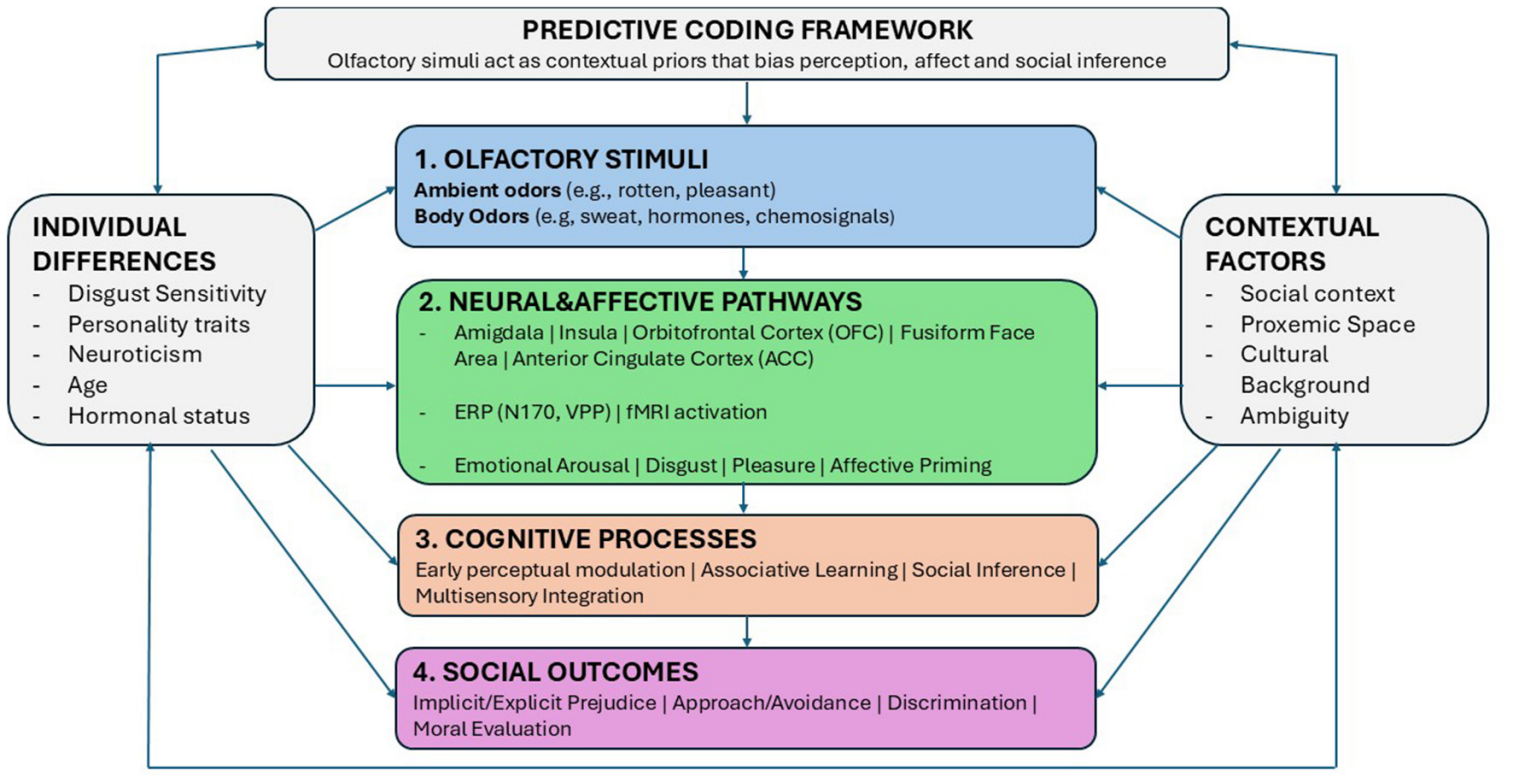
Conceptual model of olfactory influences on social cognition and prejudice. The model depicts the pathway from olfactory stimuli (ambient odors, body odors, chemosignals) through neural and affective processing to social judgments, with moderating influences of individual differences and contextual factors. Key neural regions (amygdala, insula, prefrontal cortex) and psychological mechanisms (disgust, mood, associative learning) are integrated within the framework.

To integrate these diverse findings into a coherent framework, we propose a conceptual model that traces the pathway from olfactory input to social judgment (Figure 2). This model illustrates how olfactory stimuli engage distributed neural circuits and affective pathways which in turn bias cognitive processes and inferential reasoning. These processes ultimately shape social judgments and prejudice, with effects moderated by individual differences and contextual factors. The model emphasizes different mechanisms that operate across processing levels and interact to shape social outcomes.

### 8.1 Olfactory influences as contextual priors: a computational perspective

A productive way to conceptualize the diverse effects reviewed here is through the lens of predictive coding and Bayesian inference, which frame perception as a process of integrating sensory evidence with prior expectations (Friston, 2010; Clark, 2013). From this perspective, olfactory stimuli can be understood as contextual priors—pre-existing expectations or biases that shape the interpretation of ambiguous social information. This framework helps specify the stage of processing, the conditions of operation, and the computational mechanisms underlying olfactory influences on social cognition.

Olfactory influences operate at multiple levels of the processing hierarchy. At early perceptual stages, odors modulate the initial encoding of sensory information, as evidenced by enhanced N170 or VPP components to emotional faces presented in congruent odor contexts (Syrjänen et al., 2018b; Li and Wang, 2021). At intermediate evaluative stages, odors bias the assignment of affective value to social stimuli, as shown by the systematic shift in face pleasantness ratings under pleasant vs. unpleasant odor contexts (Syrjänen et al., 2018a). At late decisional stages, odors influence higher-order judgments, such as trait attributions (Homan et al., 2017) and moral evaluations (Cecchetto et al., 2019), by shaping the interpretation of behavioral evidence in light of the emotional context.

The influence of olfactory contextual priors is not automatic or uniform but depends on several conditions. First, awareness: many effects operate subconsciously, as demonstrated by studies using subliminal odor presentation (Hummer and McClintock, 2009; Cecchetto et al., 2019), but conscious perception can amplify or modulate these effects (Cunningham et al., 2013). Second, contextual relevance: odors generate stronger effects when they are congruent with or relevant to the social context, such as the influence of androstadienone restricted by the presence of a male experimenter (Lundström and Olsson, 2005). Third, stimulus strength and ambiguity: olfactory priors are most influential when visual information is ambiguous (Kovács et al., 2004; de Groot et al., 2021), consistent with Bayesian principles that whereby priors exert their greatest influence when sensory evidence is weak or noisy.

When an individual encounters a social stimulus (e.g., a neutral or ambiguous face), the brain combines the sensory evidence (the visual input) with prior expectations derived from the current context. The olfactory context provides one such prior, effectively shifting the baseline probability that the face will be interpreted as disgusted, threatening or otherwise emotionally salient. For example, in the presence of an unpleasant body odor, the prior probability of perceiving fear or disgust in an ambiguous face is increased (de Groot et al., 2021). Importantly, this prior is not static but can be updated through learning, as demonstrated by (Gaby and Dalton 2019), who showed that individuals formed new associations between a person's odor and aversive outcomes, creating a learned prior that biased subsequent social judgments.

### 8.2 Olfactory influences through the lens of multisensory integration

The effects of odors on social perception can be understood through the framework of multisensory integration research, which has established that the brain actively integrates information across sensory channels (Stein and Meredith, 1993; Calvert and Thesen, 2004). This perspective is particularly relevant for olfaction, given its unique neuroanatomical connections and its frequent co-occurrence with visual social stimuli.

From a predictive coding perspective (already discussed above), olfactory inputs can serve as contextual priors that bias social perception, particularly when visual cues are ambiguous. For example, (de Groot et al. 2021) demonstrated that fear-related body odors biased participants toward perceiving fear in ambiguous face morphs, consistent with the notion that olfactory cues shift the perceptual decision boundary. Similarly, Li and Wang (2021) found that unpleasant odors enhanced the recognition of bodily expressions, suggesting the facilitation of emotionally congruent predictions.

Crossmodal emotion integration occurs at multiple neural levels, with odors and emotional faces converging in regions including the amygdala, insula, and fusiform gyrus (Seubert et al., 2010; Cecchetto et al., 2019). These regions exhibit enhanced activation when olfactory and visual inputs are emotionally congruent (Müller et al., 2014). Importantly, these congruency effects are not uniform: the mood-congruency effect observed by Li and Wang (2021) suggests that odors bias perception toward emotionally congruent interpretations, while (Syrjänen et al. 2018a) found that odors biased the perceived valence of faces regardless of facial expression. This pattern aligns with hierarchical models of multisensory integration, which propose that congruency effects can occur at both early (pre-attentive) and late (post-perceptual) stages, with different computational mechanisms operating at each level (Calvert and Thesen, 2004).

Finally, the olfactory system's direct projections to limbic structures support a hierarchical model in which odors can influence social cognition at multiple levels: a subcortical route involving rapid, automatic activation of affective responses; a cortical route involving conscious evaluation and cognitive appraisal; and an integrative route where olfactory and visual information combine in regions such as the orbitofrontal cortex and anterior insula. This hierarchical architecture explains why olfactory effects can be observed across diverse social processes—from rapid perceptual biases to deliberate judgment—and why the same odor can influence different outcomes depending on the processing level and context.

### 8.3 Beyond the behavioral immune system: multiple mechanisms of olfactory influence

The Behavioral Immune System (BIS) offers an explanation for some findings, particularly those linking disgust to prejudice and outgroup avoidance, but its explanatory scope is not universal. Olfactory effects on social perception also involve crossmodal integration, affective priming, associative learning, and modulation of attentional and evaluative processes that operate independently of pathogen avoidance. Pheromone-like effects (e.g., androstadienone and hedione) are unlikely to be mediated by disgust; instead, they may modulate social perception through hormonal pathways, affecting mood, attention, and social judgments independent of affective valence (Hummer and McClintock, 2009; Oleszkiewicz et al., 2021). Similarly, odor effects on emotional perception—such as the modulation of face and body expression recognition—are well explained by crossmodal integration and embodied cognition frameworks, where affective states evoked by odors “spill over” into the interpretation of ambiguous social signals through general arousal and mood mechanisms (Li and Wang, 2021; Seubert et al., 2010; Barsalou, 2008). Such effects do not require BIS activation and can occur with pleasant as well as unpleasant odors (Seubert et al., 2010). Recognizing these multiple pathways enables a more comprehensive understanding of how odors shape social cognition.

### 8.4 Critical evaluation of evidence quality

A balanced assessment requires acknowledging substantial variability in the strength and replicability of the evidence across different domains, particularly in the literature on putative human pheromones.

The effects of androstadienone on mood, attention, and social perception have been inconsistent. While some studies reported positive effects (Lundström and Olsson, 2005; Hummer and McClintock, 2009), well-powered studies failed to replicate them (Hornung et al., 2018; Lu et al., 2024). Similarly, hedione effects on attractiveness (Oleszkiewicz et al., 2021) were based on a small sample (*n* = 17) and have not been independently replicated. These inconsistencies may reflect subtle effects requiring specific conditions, or they may indicate that the effects are not robust. Null findings from large, pre-registered studies and Bayesian analyses (Lu et al., 2024; Hornung et al., 2018) suggest that the evidence for pheromone effects is weaker than sometimes claimed. Contributing factors include publication bias, variation in methodologies, differences in participant selection, insufficient statistical power, and the absence of pre-registration.

In contrast, evidence for ambient and body odor effects on social perception and prejudice is more consistent. Studies on disgust-induced prejudice (Inbar et al., 2012; Cunningham et al., 2013), body odor disgust sensitivity and xenophobia (Zakrzewska et al., 2019, 2020), and odor effects on face perception (Syrjänen et al., 2018a,b) have generally converged across independent samples and approaches. However, even in this domain, larger sample sizes, more diverse populations, and ecologically valid paradigms would strengthen the external validity.

This idea of context-specific modulation is crucial for understanding compounds such as androstadienone and Hedione, the findings lend themselves to interpretation through the Emotional Attention Hypothesis (Hummer and McClintock, 2009). According to this hypothesis, these compounds do not act as simple “pheromones” in the classical sense (i.e., as signals triggering stereotyped behavioral responses), but rather as fine-tuned modulators that tune the attentional system toward emotionally salient information. The data from (Hummer and McClintock 2009) directly support this hypothesis: androstadienone accelerates reactions to emotional faces in a dot-probe task and slows color-naming for emotional words in an emotional Stroop task, but has no effect on non-emotional or non-social stimuli. This specificity profile indicates that the compound selectively acts on emotional attention circuits, not on general or social attention.

Parallel to this, the study by (Lundström and Olsson 2005) adds a crucial layer of complexity: the positive effect of androstadienone on women's mood and physiological arousal occurs only when the experimenter is male. This finding is perfectly interpretable through the lens of socio-constructivist theories and situated cognition (Smith and Semin, 1993), which emphasize how cognitive processes are always situated in a physical and social context. The social meaning of the chemosignal—and therefore its effect—emerges from the interaction between the stimulus and the relational context in which it is perceived.

This context-dependency is further elaborated by studies on associative learning, which demonstrate that the significance of an olfactory stimulus is not fixed but can be actively shaped by experience. The classical conditioning paradigm used by (Gaby and Dalton 2019) demonstrates that body odors can serve as conditioned stimuli, acquiring negative valence through association with a shock. This finding aligns with models of Pavlovian conditioning applied to chemoreception and shows that the olfactory system is finely tuned to learn threat associations specific to individuals. Of particular interest is that this learning persists even when the odor is masked by perfume, suggesting that perfumes do not erase the individual's “olfactory signature.” From an evolutionary perspective, this characteristic has clear adaptive value: it allows us to maintain traces of negative experiences with specific individuals even in contexts where body odor is altered by cosmetic products. Furthermore, the fact that this learned aversion “spills over” to influence the perception of neutral faces—rated as more surprised in the presence of the shock-associated odor—demonstrates that olfactory learning does not remain confined to the sensory domain but extends to influence social cognition in cross-modal ways. This phenomenon can be interpreted through predictive coding models (Friston, 2010), according to which the brain constantly generates predictions about incoming stimuli based on past experiences: the odor predicts a threat, and this prediction alters face perception, making it appear more “surprised” and therefore potentially more reliable as an alert signal. Taken together, these findings across theoretical frameworks illustrate how the olfactory system, through a combination of evolved predispositions, embodied neural mechanisms, context-dependent attentional modulation, and associative learning, serves as a powerful and often hidden driver of our social perceptions and interactions.

In summary, the present findings suggest that olfactory influences on social cognition emerge from the interaction of multiple mechanisms, including disgust-related processes, embodied affective integration, context-dependent modulation, and associative learning. Importantly, these effects are not uniform but are constrained by individual differences, contextual relevance, and the strength of the olfactory signal, highlighting important boundary conditions for their generalization.

### 8.5 Theoretical and practical implications

The integrated framework developed in this review carries several important implications for both theoretical models of social cognition and practical applications.

Our findings challenge dual-process models that distinguish between automatic and controlled processing in social cognition. Olfactory influences operate across a continuum of processing stages, from early perceptual biases to later deliberate evaluations, with dynamic interactions between these levels. This aligns with integrative accounts emphasizing continuity between perception, affect, and higher-order cognition (Cunningham and Zelazo, 2007; Barrett, 2017). Furthermore, the concept of olfactory contextual priors extends predictive coding models to the social domain, proposing that the brain incorporates multisensory contextual information—including olfactory signals—to optimize social inference. This perspective suggests that social cognitive theories should consider the sensory context in which social judgments occur, rather than treating social perception as purely visual or conceptual.

The findings have potential applications across several real-world contexts. In educational and workplace settings, awareness of ambient odor effects on social evaluations could inform environmental design to minimize bias—for instance, maintaining neutral or pleasant odors in hiring or assessment contexts may reduce evaluative biases. In clinical contexts, understanding how olfactory disgust sensitivity contributes to prejudice and social avoidance could inform interventions targeting social anxiety, xenophobia and related forms of social bias. The use of olfactory stimuli in virtual reality-based interventions (Invitto et al., 2025) offers novel opportunities to study and modulate implicit biases in controlled yet ecologically valid environments. Finally, considering olfactory factors in designing public and diverse environments could contribute to reducing implicit prejudice and promoting social cohesion.

### 8.6 Limitations and future directions

Several limitations in the current literature warrant attention. First, many studies rely on laboratory-based paradigms with limited ecological validity, which may not capture the complexity of real-world multisensory environments. The effects of odors, especially chemosignals, are often subtle and contingent on context, sample characteristics, and methodological specifics, leading to variability and inconsistencies across studies (e.g., Lu et al., 2024 vs. Oleszkiewicz et al., 2021). Second, there is considerable methodological heterogeneity, including variability in odor delivery systems, concentration levels, and control conditions, which limits comparability across studies. Third, individual differences (e.g., disgust sensitivity, personality traits, and biological factors) are not consistently assessed or controlled, despite evidence suggesting their moderating role.

Furthermore, the literature search was limited to two databases (PubMed and Web of Science). Although these databases provide broad coverage of biomedical, psychological, and multidisciplinary research, additional sources such as PsycINFO and Scopus may have identified further relevant studies and increased the comprehensiveness of the evidence base.

In addition, because of the substantial heterogeneity of the included studies in terms of experimental paradigms, olfactory stimuli, outcome measures, and study designs, a formal quantitative synthesis (meta-analysis) was not considered appropriate. Likewise, although study quality was systematically evaluated using predefined qualitative criteria applied independently by two reviewers, no standardized risk-of-bias tool was employed. Consequently, the findings should be interpreted with appropriate caution, particularly when comparing evidence across different experimental paradigms.

Nevertheless, this review was conducted using a systematic search strategy, predefined eligibility criteria, and a transparent study selection process following the PRISMA 2020 framework. The available evidence was therefore synthesized using a narrative approach to account for the marked methodological heterogeneity of the included studies.

Future research should prioritize more ecologically valid approaches, such as the pioneering protocol proposed by Invitto et al. (2025), which combines virtual reality (VR) with olfactory stimuli and neuroimaging (EEG) to study proxemic behavior and implicit bias in realistic social scenarios. This will be crucial for understanding how odors operate in the complex, multi-sensory contexts of real life. Further investigation is also needed into how olfactory influences change across the lifespan, as suggested by the age-related dedifferentiation of emotional responses to smells (Vieillard et al., 2021), and how cultural factors shape the intersection of odor, disgust, and social judgment.

## 9 Conclusion

This systematic review synthesizes evidence indicating that olfactory stimuli can modulate human social cognition and intergroup evaluation in subtle and context-dependent ways. Odors, particularly those eliciting disgust, can amplify prejudices and bias social perceptions, largely operating through subconscious pathways linked to the Behavioral Immune System. Putative human chemosignals, when effects are observed, seem to modulate attention and evaluative processes in a context-dependent manner. These processes are supported by distributed neural systems, including limbic and insular regions involved in affective and interoceptive processing.

Critically, these processes are not uniform but vary as a function of individual differences (e.g., disgust sensitivity), contextual relevance, and task demand. Overall, the findings suggest that olfactory inputs represent a relevant, yet often overlooked, component of social cognition. Integrating olfactory processing into models of social cognition may therefore contribute to a more comprehensive understanding of human social behavior. Future research adopting ecologically valid and multimodal approaches (e.g., Virtual Reality paradigms) will be important to clarify how olfactory cues interact with other sensory and social signals, and under which conditions they influence social evaluation and bias.

## Data Availability

The original contributions presented in the study are included in the article/supplementary material, further inquiries can be directed to the corresponding author.
